# The New Frontiers of Fetal Imaging: MRI Insights into Cardiovascular and Thoracic Structures

**DOI:** 10.3390/jcm13164598

**Published:** 2024-08-06

**Authors:** Giulia Cundari, Nicola Galea, Daniele Di Mascio, Marco Gennarini, Flavia Ventriglia, Federica Curti, Martina Dodaro, Giuseppe Rizzo, Carlo Catalano, Antonella Giancotti, Lucia Manganaro

**Affiliations:** 1 Department of Radiological, Oncological and Pathological Sciences, Sapienza University of Rome, Policlinico Umberto I, Viale Regina Elena 324, 00161 Rome, Italy; giulia.cundari@uniroma1.it (G.C.); nicola.galea@uniroma1.it (N.G.); marco.gennarini@uniroma1.it (M.G.); federica.curti@uniroma1.it (F.C.); martina.dodaro@uniroma1.it (M.D.); carlo.catalano@uniroma1.it (C.C.); lucia.manganaro@uniroma1.it (L.M.); 2 Department of Maternal and Child Health and Urological Sciences, Sapienza University of Rome, Policlinico Umberto I, Viale Regina Elena 324, 00161 Rome, Italy; daniele.dimascio@uniroma1.it (D.D.M.); flavia.ventriglia@uniroma1.it (F.V.); giuseppe.rizzo@uniroma1.it (G.R.)

**Keywords:** fetal magnetic resonance, fetal cardiac magnetic resonance, fetal cardiac gating, congenital heart disease, 4D flow images, congenital chest pathologies

## Abstract

Fetal magnetic resonance imaging (fMRI) represents a second-line imaging modality that provides multiparametric and multiplanar views that are crucial for confirming diagnoses, detecting associated pathologies, and resolving inconclusive ultrasound findings. The introduction of high-field magnets and new imaging sequences has expanded MRI’s role in pregnancy management. Recent innovations in ECG-gating techniques have revolutionized the prenatal evaluation of congenital heart disease by synchronizing imaging with the fetal heartbeat, thus addressing traditional challenges in cardiac imaging. Fetal cardiac MRI (fCMR) is particularly valuable for assessing congenital heart diseases, especially when ultrasound is limited by poor imaging conditions. fCMR allows for detailed anatomical and functional evaluation of the heart and great vessels and is also useful for diagnosing additional anomalies and analyzing blood flow patterns, which can aid in understanding abnormal fetal brain growth and placental perfusion. This review emphasizes fMRI’s potential in evaluating cardiac and thoracic structures, including various gating techniques like metric optimized gating, self-gating, and Doppler ultrasound gating. The review also covers the use of static and cine images for structural and functional assessments and discusses advanced techniques like 4D-flow MRI and T1 or T2 mapping for comprehensive flow quantification and tissue characterization.

## 1. Introduction

The application of magnetic resonance (MRI) in fetal imaging has been impacted by technological advancements in the previous 20 years. Fast imaging techniques allowed the possibility to examine fetal anatomy by solving the issue of image motion artifacts brought on by fetal movements [1]. Fetal MRI (fMRI) represents a second-line imaging modality performed after II-level prenatal ultrasound; due to its intrinsic properties (multiparametric and multiplanar examinations), fMRI is a useful diagnostic tool to confirm diagnosis, to evaluate the presence of associated pathologies and to assess inconclusive ultrasound (US) examination. Indeed, thanks to technological advancements, such as the introduction of a 3.0 T magnet and the development of new sequences, including the use of diffusion-weighted images [2,3,4], MRI plays an increasing role in the management of pregnancy [5]. The most frequent use of fMRI imaging is represented by the evaluation of central nervous system anomalies; however, fMRI is also useful for assessing fetal body pathologies, such as chest and abdominal malformations [6].

Regarding the study of fetal heart disease, a great impact has been given by new methods of ECG-gating, which have been recently introduced in the routine MRI examination [7]. With these techniques, which will be discussed in the present review, fetal heartbeat can be synchronized with sequences’ acquisition, thus overcoming traditional drawbacks related to cardiac imaging in this population of patients. Fetal cardiac MRI (fCMR) could represent an important diagnostic tool in the assessment of congenital heart disease or when US faces limitations like poor acoustic windows due to factors such as maternal obesity, oligohydramnios, fetal lie, or calcified ribs. fCMR can evaluate the anatomy and the function of the heart and great vessels, also providing the ability to detect additional anomalies; moreover, by analyzing blood flow patterns, fCMR represents a potential tool for the diagnosis of abnormal fetal brain development and placental perfusion [8].

This review will focus on the potential applications of fMRI in the assessment of cardiac and thoracic pathologies. An overview of fCMR protocols and cardiac gating technique, as well as a discussion of the potential application of fMRI in cardiac and thoracic pathologies, is offered to explore current trends and emerging applications.

## 2. Chest MRI

### 2.1. Imaging Acquisition

**Figure 1:**
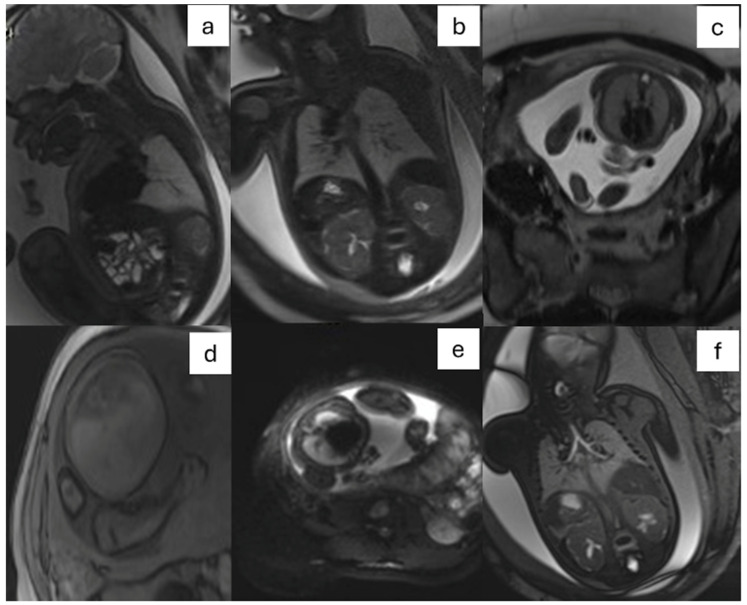
Fetal chest MRI protocol: T2-weighted single-shot fast spin echo in sagittal, coronal, and axial (**a**–**c**) planes; T1-weighted three-dimensional (3D) GRE on axial plane (**d**); DWI EPI (**e**) on axial plane; balanced steady-state-free precession on coronal plane (**f**). DWI: diffusion-weighted imaging; EPI: echo planar imaging; GRE: gradient echo; MRI: magnetic resonance imaging.

The International Society of Ultrasound in Obstetrics and Gynecology recommends T2-weighted sequences in three orthogonal planes of the fetal brain and body and T1-weighted and gradient-echo sequences in one or two orthogonal planes [9]. Our proposed 3-T MRI protocol includes T1-weighted 3D gradient echo (GRE) and T2-weighted fast spin echo (FSE) sequences in axial, sagittal, and coronal planes. The intravoxel incoherent motion (IVIM) protocol incorporates diffusion-weighted imaging—echo planar (DWI-EPI) sequences with diffusion-encoding gradients along three non-coplanar directions and multiple b-values (Figure 1, Table 1).

- T1-weighted 3D GRE: T1 contrast optimization at 3 T involves adjusting repetition time (TR), echo time (TE), and flip angle, with fat-saturated T1 3D GRE Dixon sequences used. Parallel imaging combined with T1-weighted 3D GRE sequences allows acquisitions during breath-holding to minimize fetal motion artifacts.
- Single-shot fast spin-echo (SS-FSE): A preferred technique for fetal imaging is the free-breathing T2-weighted SS-FSE sequence, despite susceptibility to fetal motion artifacts. Dielectric artifact, influenced by body region and patient physiology, is a limitation at 3 T. The artifact worsens with larger maternal diameters and amniotic fluid volumes. Mitigation techniques include saturation bands, increased flip angle, and prescan-B1 filter application. However, these may increase radiofrequency power deposition and require longer TR. Adjusting fetal positioning relative to the body coil can optimize artifact reduction.
- Balanced steady-state-free precession (bSSFP): bSSFP sequences offer a high signal-to-noise ratio and T2/T1 image contrast, beneficial for heart and vessel evaluation due to bright-blood signal. Adjusting offset frequency mitigates banding artifacts, altering image contrasts. Real-time bSSFP imaging captures cardiac motion, ideal for uncooperative patients, achieving temporal resolution of 1.5 phases [5].

Among the advantages of using 3T magnets is the greater signal-to-noise ratio that translates into faster image acquisition, which allows for the reduction in fetal movement artifacts.

The increase in the magnetic field has the side effect of increasing the Specific Absorption Rate (SAR) and making image acquisition more susceptible to chemical shift artifacts, B1 inhomogeneities, and standing waves [5].

The use of 3T magnets is not possible in all diagnostic centers, and a recent investigation shows that in Europe only 30% of centers perform fMRI on 3T scanners [10].

In the table below, our suggested protocol for performing the exam on a 1.5T scanner (Table 2).

### 2.2. Clinical Application

The indication for further diagnostic investigation with an MRI examination of the fetal chest is justified by an accurate delineation of lung anatomy and morphology in T2-weighted sequences. MRI can distinguish between normal and abnormal lung tissue and helps in several diagnoses recognition—such as congenital diaphragmatic hernia (CDH)—especially in later gestational stages better than US. In addition, with DWI acquisitions, it is possible to study the degree of lung maturity given the evidence that the apparent diffusion coefficient (ADC) value increases with advancing gestational age (GA).

The examination is proposed in fetuses with congenital chest mass, congenital pulmonary airway malformation, and congenital diaphragmatic hernia as these conditions are associated with pulmonary hypoplasia. Generally, lung lesions are asymptomatic during fetal development, but larger lesions can lead to hydrops, caused by compression of adjacent structures, with poor prognosis if left untreated.

In bronchopulmonary malformations, fMRI can provide information on lung volume and shows indirect signs of parenchymal function through the T2 signal. It also allows proper assessment of airway patency, helping in the selection of patients who are candidates for the EXIT (EX utero Intrapartum Therapy) procedure.

Pulmonary evaluation by MRI should be proposed in fetuses with CDH, congenital pulmonary airway malformation (CPAM), or congenital thoracic masses, which are often associated with pulmonary hypoplasia [12,13,14,15]. Among the many possible methods proposed to measure fetal lung volumes on MRI [16,17,18], the most common is to draw a region of interest (ROI) around the residual lung, most frequently in the axial plane [12,19,20], and then multiply it by the slice thickness. Predicted percent lung volume (PPLV) is useful to avoid wide variations in normal lung volume measurements [18].

However, the most recognized approach to determine the outcome of fetuses with CDH is the ratio of measurements, either the observed fetal lung volume (oFLV) or the expected fetal lung volume (eFLV) [12,21]. Some authors have proposed standardized measurements of lung volume and its correlation with outcomes for other thoracic abnormalities such as CPAM, creating the CPAM volume ratio (mass volume divided by fetal head circumference), based on ultrasound measurements [22], and the MRI lung mass volume ratio [18,23].

**Figure 2:**
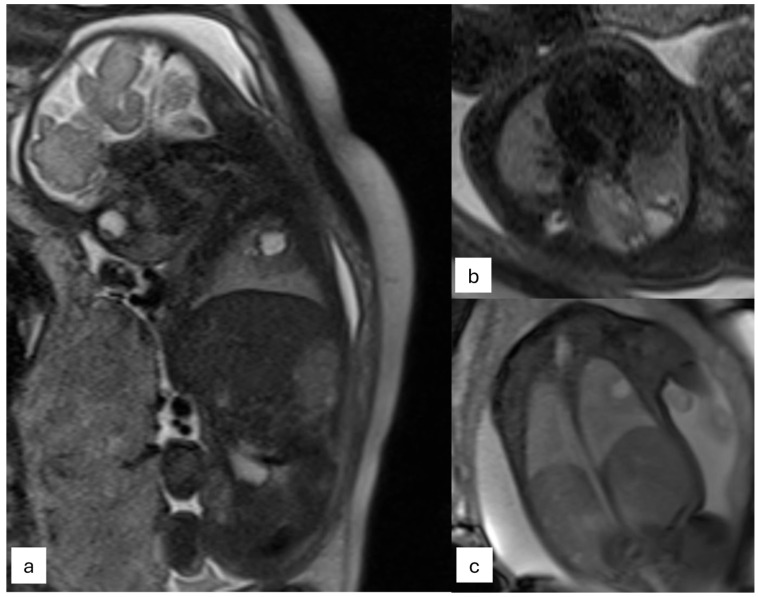
Examination performed on a 1.5 T magnet. T2 sagittal (**a**) and axial plane (**b**), Trufi coronal plane (**c**). The fetus of 32 gestational weeks with type II cystic adenomatosis. At the level of the right upper lobe, non-homogeneous signal intensity is observed due to the presence of some medium and small cystic formations (diameter 10 mm) located mainly in the peripheral area.

**Figure 3:**
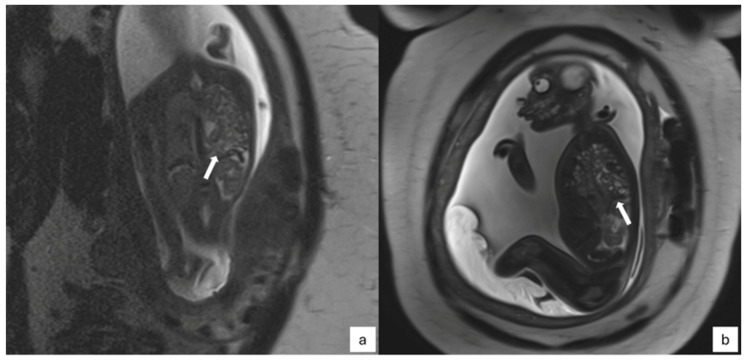
T2-weighted half-Fourier acquisition single-shot turbo-spin echo (HASTE) images of the same fetus with a congenital diaphragmatic hernia (white arrows) in different gestational ages on coronal planes ((**a**)—23 weeks of gestation) and sagittal planes ((**b**)—31 weeks of gestation).

The most common thoracic pathologies are as follows:

- CPAM: This is the most frequent malformation, consisting of communication with the normal tracheobronchial tree. They can present in solid or cystic form and are classified into 5 subcategories (Figure 2).

- Bronchopulmonary sequestration (BPS): It is the second most frequent cause of lung disease, characterized by the presence of lung parenchyma not communicating with the tracheobronchial tree. BPS is classified into two categories, extra-lobar or intra-lobar, depending on the presence of an independent pleura.
- Congenital lobar overinflation (CLO): It represents hyperinflation of a lung segment or lobe caused by bronchial obstruction.
- Congenital bronchogenic cyst: A fluid-containing lesion with thin walls often located near the carina.
- Congenital upper airway obstruction syndrome (CHAOS): It is caused by obstruction of the larynx or trachea from an extrinsic or intrinsic cause, resulting in fluid entrapment in the lungs and dilatation of the trachea.
- Bronchial atresia: This malformation results in secondary changes to the distal lung parenchyma that appears homogeneously hyperintense in T2-weighted sequences. It is subcategorized into two groups, proximal type and peripheral type.
- CDH: It is defined as a herniation of abdominal organs in the thorax through an orifice of the diaphragm caused by delayed or abnormal separation of the thoracic and abdominal compartments (Figure 3).

The most common are posterolateral (70–75%), and most frequent on the left side (85%). The amount of normal residual lung is a key question for imaging to address in prenatal counseling. Thus, regarding the applications of MRI in CDH, this technique has demonstrated an added value in the identification, localization (right or left), and evaluation of herniated organs, particularly the bowel, liver, and kidneys. Also, MRI was found to be useful in providing information on lung volume, allowing the measurements of residual and contralateral lung, and providing indices of fetal lung maturity with analysis of lung signal intensity. The evaluation of these parameters, combined with the assessment of mediastinal shift, the presence or absence of polyhydramnios, fetal hydrops, and other conditions that may be associated, allows for the identification of high-risk fetuses (with liver herniation or delayed lung maturation) that would benefit from Fetoscopic EndoTracheal balloon Occlusion (FETO) in utero and EXIT procedure at birth. Fetuses considered low-risk, that is, with a liver in place and normal lung parameters, may be candidates for elective postnatal reduction surgery [11]. 

## 3. Cardiac Magnetic Resonance Imaging

### 3.1. Fetal Cardiac Gating

In order to obtain diagnostic images of the fetal heart, it is necessary to synchronize sequences’ acquisition to the fetal heartbeat. Several cardiac gating techniques have been so far developed: metric-optimized gating (MOG), self-gating, and Doppler ultrasound (DUS) gating.

**Figure 4:**
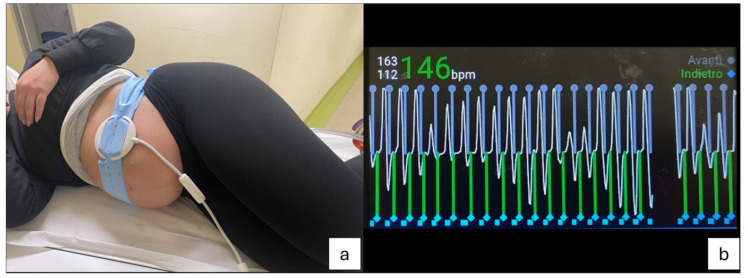
(**a**) Doppler ultrasound device positioned and fastened on the maternal abdomen in lateral decubitus and (**b**) fetal heartbeat track obtained with the DUS method.

Using a mathematical model of the cardiac cycle, MOG reconstructs images of the fetal heart, by reducing image entropy in time, space, or both, thus maximizing image quality. It can be used with various MRI acquisition techniques and k-space filling methods, such as radial and Cartesian bSSFP. One of the distinctive characteristics of MOG is that it parameterizes each fetal cardiac trigger separately. This indicates that it considers the fetal heart rate’s beat-to-beat variations when reconstructing the images. However, major drawbacks of MOG deal with the oversampling of the k-space matrix, which necessitates a lengthy acquisition time, the vulnerability to heart-rate variability, and the long post-processing elaborations [24,25]. Regarding the self-gating technique, heartbeat signals are taken straight out of the MRI scan. In fact, it eliminates the need for extraneous hardware or sensors by enabling the MRI system to autonomously select when to acquire images during specific cardiac or breathing cycle phases. While self-gating can be applied to a variety of k-space trajectories, such as Cartesian, radial, and spiral, it has primarily been employed to acquire radial data in the setting of fMRI. Nonuniform clustering of data following temporal sorting, k-space trajectory errors, and off-resonance artifacts are some of the problems that must be handled using non-Cartesian sampling techniques [25,26]. DUS cardiac gating is an accurate and high-quality method to synchronize ECG signals with MRI acquisition. With this technique, the DUS device is placed on the maternal abdomen (both in the supine or lateral decubitus [27,28]) and fastened using an elastic belt. The transducer and the cable, which are composed of non-magnetic materials, transmit DUS signals toward the fetal heart by piezoelectric crystals within the transducer [27,28,29]. The pulses are reflected by the flowing blood and the beating cardiac tissues; after the amplification and filtering of the pulses, signal peaks are sent to the MRI unit, coding the reference systolic wave through which the cardiac cycles are identified [30,31,32,33] (Figure 4). This technique allows for saving time for further post-processing analysis. The main limitations related to the DUS gating method deal with the loss of cardiac signal due to fetal movements or deep maternal inspiration during the examination; therefore, fCMR with DUS is usually performed in the third trimester (fetal movements become less frequent with the growing fetus because of increased occupancy of the uterus) [26]. Other drawbacks of the DUS cardiac gating technique are the mother’s high body mass index or the placenta’s location on the anterior portion of the uterine wall: In those cases, the bad acoustic window can prevent the acquisition of a sufficient gating signal. 

### 3.2. Image Acquisition

#### 3.2.1. Cardiac Planes

Black blood sequences, such as SS-FSE or half-Fourier acquisition single-shot turbo-spin echo (HASTE), are used for static imaging of the fetal heart [34].

**Figure 5:**
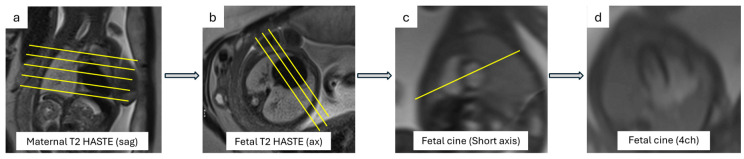
Cardiac planes. The figure shows how to get specific fetal cardiac planes (yellow lines demonstrate how to orient acquisition planes): (**a**) T2 HASTE of the maternal sagittal plane; (**b**) T2 HASTE scan perpendicular to the fetal thorax to obtain fetal axial planes; (**c**) cine scan perpendicular to the interventricular septum to obtain short axis fetal cine; (**d**) a real 4-chambers view can be then found.

Before cardiac sequences are acquired, these sequences can be used to show transversal, coronal, and sagittal views of the fetal thorax [27,28,29,33]. These static sequences, which have a low acquisition time and high spatial resolution, enable the detection of mediastinal vascular abnormalities, accurate representation of macroscopic anatomy, and entire fetal body evaluation for extracardiac defects. Static sequences have the disadvantage of only offering broad anatomical information but do not provide functional data or detailed morphology [34] (Figure 5). 

Often, these sequences are used as a reference for cardiac plane preparation and serve as a propaedeutic approach to morpho-functional evaluation with cine images. Cardiac planes can be prepared as the standard axial, sagittal, and coronal body planes (the fetal lung is not inflated, so the heart appears more horizontal, and the axial plane resembles a 4-chamber view) or as the specific cardiac planes also used for adult heart evaluation, which offer a more precise assessment of heart volumes and function [7].

#### 3.2.2. Cine Images

**Figure 6:**
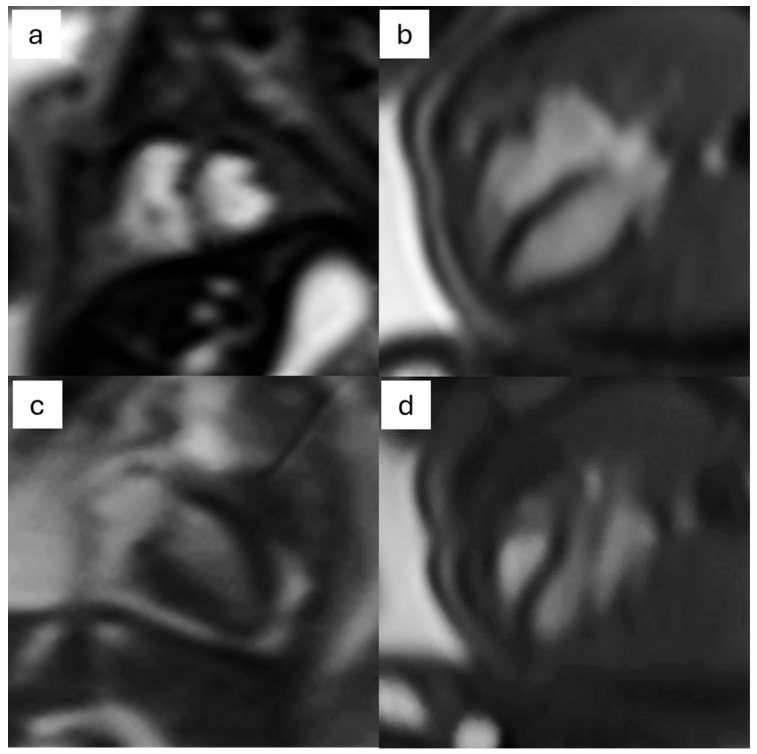
Cine images of the fetal heart: (**a**) short axis, (**b**) 4-chambers view, (**c**) 2-chambers view, and (**d**) 3-chambers view.

Cardiac and vascular anatomy, together with cardiac functional assessment, are portrayed through the acquisition of cine images throughout the cardiac cycle [35] (Figure 6). The ventricular and atrial structures, as well as the papillary muscles, the foramen ovale, the atrial and ventricular septa, the atrioventricular valves, the semilunar valves, the major arteries, the systemic veins, and the pulmonary veins, are all defined by long axis and short axis views [27,36,37,38]. Cine images can be therefore used to evaluate a number of congenital abnormalities, including hypoplastic left heart syndrome, pulmonary atresia, atrioventricular septal defect, and aortic arch deformities [33,38]. Significant reference values were provided by Minocha et al. [39] for fetal cardiac dimensions, volumes, and function; their results showed that there was a 48% difference in volumes when compared to echocardiography. Major difficulties with cine image acquisition concern the small dimensions of cardiac and vascular fetal structures along with the large field of view, which is required to increase signal-to-noise ratios (SNRs) and to avoid wrap-around aberrations from the mother’s body. This is especially difficult in obese expectant mothers or in cases of polyhydramnios, which further lowers the SNR [40].

#### 3.2.3. Flow Imaging

Phase-contrast MRI (PC-MRI) is the reference method for postnatal hemodynamic assessment in children with congenital heart disease. It is a powerful technology that allows the non-invasive blood flow quantification and the assessment of cardiac output in the left and right ventricles [41,42]. Additionally, these sequences represent a reliable method for assessing fetal aortic or pulmonary flow, atrioventricular valve dynamics, and the existence of interventricular or interatrial shunts. Adult sequence planes function in the same way [29]. It has been demonstrated that PC-MRI is valid in quantifying the flow in the main vessels of the fetus late in gestation, thanks to the technique of MOG [41]. The principal limitations of PC-MR are the long acquisition time and the limited flow information at the plane of interest [41].

Four-dimensional flow MRI (4D flow MRI), enabled by the acquisition of three-dimensional PC-MRI sequences, is an appealing technology that provides a comprehensive assessment and evaluation of blood flow vectors of a large three-dimensional volume in a single acquisition. 

**Figure 7:**
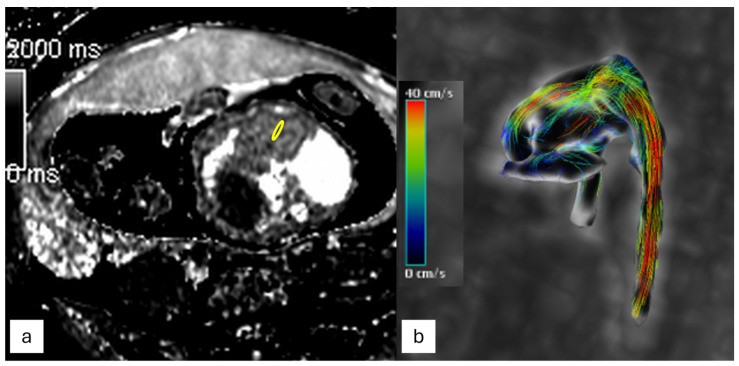
(**a**) T1 mapping of the fetal heart in 4-chambers view: a T1 myocardial value of 1726 ms was found, by tracing an ROI on the myocardial interventricular septum (yellow circle) and (**b**) 4D-flow imaging of the thoracic aorta of the fetus.

The creation of sequences for 4D flow assessment—that is, flow in three dimensions with time as the fourth dimension—is an interesting new advance in fetal CMR [41], which offers the benefit of a quicker and more thorough evaluation [43]. Time-resolved sets of 3D volumes during a cardiac cycle are provided by 4D flow MRI, which also allows for the co-registration of morphologic images with flow data. Within the obtained volume, 4D flow MRI offers comprehensive flow analysis in each vascular or cardiac plane and provides additional flow parameters, such as forward and backward flow volumes, flow velocities, and shunt volumes [44], including the depiction of flow disturbances. Furthermore, 4D flow MRI sequences might be clinically more helpful than fetal echocardiography since the acquisition is not dependent on the fetal intrauterine location. However, the acquisition is susceptible to cardiac gating signal loss due to fetal movements during image acquisition [27,28] (Figure 7).

#### 3.2.4. T1 and T2 Mapping

Another special feature of CMR is its capacity to non-invasively measure blood oxygenation. The hematocrit of blood within a vessel and oxygen saturations can both be determined by using T1 and T2 relaxation times. By utilizing the Fick equation in conjunction with PC-MRI flow data, oxygen delivery may be accurately and practically assessed. With consequences for brain development and growth, fetal CMR has provided significant insights into the circulations of fetuses with congenital heart disease [42] (Figure 7).

### 3.3. Clinical Applications

Main indications for fCMR include maternal obesity, oligohydramnios, and other conditions, in which fetal US scan may be non-diagnostic [8]. 

In particular, the diagnostic pathway always starts with a screening morphological US scan; in case of maternal risk factors or suspected CHD, fetal echocardiography is then performed (risk factors include pregestational diabetes mellitus (DM) or DM diagnosed in the first trimester; uncontrolled phenylketonuria, SSA/SSB+ autoantibodies with a previously affected child; CHD in a first-degree relative of the fetus (maternal, paternal, or sibling) or specific maternal medication use [45]). In case of unclear prenatal diagnosis by echocardiography, fCMR is required.

The most frequent congenital heart diseases (CHDs) in fetuses are represented by septal defects and coarctation of the aorta [11]. The added value of performing an fCMR as a second-line imaging modality is supported by its ability to significantly improve prenatal and post-natal management of the patients. As an example, fCMR can be used to assess aortic arch and pulmonary veins anatomy, as well as to investigate extracardiac anomalies that are frequently associated with CHD or to evaluate cardiac masses [35,46]. 

Thanks to the intrinsic physic properties of CMR sequences, the tissue composition of intracardiac masses can be characterized and differential diagnosis can be performed, allowing for an eventual in utero treatment (i.e., administration of Sirolimus in patients with cardiac rhabdomyoma) [35,47]. Table 3 summarizes the main clinical indications of fCMR (Table 3). 

Fetal echocardiography might be quite limited in the evaluation of great vessels, such as in cases with tetralogy of Fallot, pulmonary valve atresia, pulmonary artery discontinuity, or aortopulmonary collateral arteries. In those patients, CMR performs well in identifying cardiac and vascular anatomy and helps in modifying patient treatment in utero or immediately after the birth [34]. In this regard, the application of 4D flow MRI sequences on a population of 16 fetuses with a gestational age of 30 + 4–38 + 5 weeks, with and without CHD, successfully allowed for comprehensive visualization and quantification of hemodynamics in the fetal great thoracic vessels [27]. Diameters of the aortic isthmus were concordant for MRI and echocardiography with a variability of 10.8% between the two techniques, in a population of 19 fetuses (mean gestational age 32.3 weeks) using the DUS gating method, reaching good diagnostic image quality and good interobserver variability [28].

Ryd et al. found that fCMR was able to improve the evaluation of patients with suspected aortic arch anomalies in 80% of their patient population, to assess the anatomy and cardiac function in 87%, and to modify patient management in 84% [38]. Vollbrecht et al. showed that fCMR was able to detect an anomalous origin of the right pulmonary artery from the ascending aorta in a patient with suspected type A interrupted aortic arch with type II aortopulmonary window. Using fCMR for surgical planning, a total repair with reconstruction of the aortic arch was performed 5 days after birth [48].

**Figure 8:**
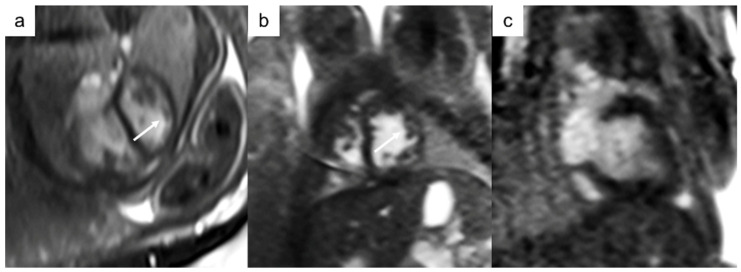
Thirty-week gestational age fetus with suspected left ventricular (LV) non-compaction syndrome at fetal echocardiography. fCMR confirmed the hypertrabeculation of the LV myocardium (white arrows) together with an increased cardio-thoracic ratio: (**a**) 4-chamber view, (**b**) short-axis view, mid-ventricular planes, and (**c**) 2-chamber view.

fCMR has the potential to offer a comprehensive evaluation of cardiac volumes and function, the quantification of aortic and pulmonary flow, and the estimation of shunts and regurgitation fraction, which are not influenced by the operator expertise (such as fetal US) or by maternal conditions that hamper fetal evaluation [34]. A study performed on fetuses with cardiac MRI at third trimester gestational age showed that fCMR assessment of anatomy and CHD reflected fetal echocardiographic findings with great accuracy [28] and with excellent image quality [36] (Figure 8). Dargahpour et al. evaluated CMR myocardial strain with feature tracking performed in a population of 38 fetuses with a gestational age of 28–41 weeks, demonstrating that strain parameters were able to differentiate between fetuses with and without CHD [49]. CHD detection rate proved a fCMR sensitivity of 91.8% and a specificity of 99.9%, using post-natal transthoracic echocardiography as the reference standard, in a population of 23 fetuses [33]. 

fCMR demonstrates several drawbacks that necessitate additional research to establish improvement: the small size of fetal anatomy (vascular structures dimensions range from 5 to 10 mm and cardiac ventricles from 10 to 30 mm) with a large field of view (FOV) required to avoid aliasing artifacts from maternal body structures (this can lead to low signal-to-noise ratio); the need for a fetal ECG gating; movement artifacts associated with maternal respiration and stochastic fetal movements, which cause shifts in the plane of acquisition [32]. fCMR is still confined to the third trimester, and despite significant postprocessing improvements, the movement artifacts can be difficult to overcome. The research process and post-processing are still time-consuming and tough to conduct. Furthermore, the technique is still quite expensive, and only specialized centers can perform adequate evaluation of CHD in such a population of patients [50]; those limitations prevent its application in low- and middle-income countries.

Future studies in this regard are needed to find sustainable solutions to improve access and efficacy of fCMR globally.

Regarding fCMR contraindications, this technique is characterized by several possible risks that must be overcome in order to reduce the risk of damage to the fetus. The main possible adverse outcomes are represented by teratogenic effects, tissue heating, and acoustic damage; however, so far no adverse consequences have been reported in the literature following a non-contrast MRI examination during pregnancy [51]. In order to avoid tissue heating and the possibility of causing malformations to the fetus, the International Electrotechnical Commission recommends not to exceed a SAR of 2 W/kg in pregnant women (this way the maternal body temperature can increase, with negligible effects on the center of the body, where the fetus lies) [52]. Regarding acoustic damage, 90 dB is the maximum acoustic noise that can be tolerated by the fetal ear before causing permanent damage. Since the maternal body cushions noises by at least 30 dB, and the maximum acoustic noise delivered by MRI scanners is around 120 dB, the possibility of acoustic damage to the fetus should not represent a concern [51,52].

## 4. Conclusions

fMRI continues to emerge as a unique non-invasive method for the prenatal evaluation of a broad spectrum of anomalies, with excellent diagnostic accuracy. fMRI transcends ultrasound limitations in challenging scenarios, improving clinical management and perinatal outcomes through detailed evaluations of chest and cardiovascular structures. 

The recent development of cardiac gating techniques and innovation in fMRI sequence are opening new frontiers in anatomical and functional evaluation, including hemodynamic assessment of blood flows.

## 5. Future Directions

Recent advancements in fMRI include the use of 3D reconstructions for congenital diaphragmatic hernia and AI-enhanced automation for faster and more accurate organ segmentation, together with innovations like real-time motion correction algorithms to reduce motion artifacts, to ameliorate the image quality, and to shorten the overall exam duration. Additionally, radiomics, thanks to its ability to derive quantitative data from MRI images, supports the identification of subtle tissue characteristics, demonstrating great potential in areas like lung volume analysis for neonatal prognosis and placental assessments to predict fetal growth limitations [5].

Using hemodynamic data not normally accessible through fetal echocardiography, fCMR approaches have been developed to measure blood flow as well as intravascular and intracardiac blood oxygen saturation. Blood oxygen measurement and flow quantification enable the application of fetal cardiac physiology information and offer insights into the effects of cardiac disease on fetal circulation, brain perfusion, and oxygen distribution [34].

## Figures and Tables

**Table 1:** Suggested 3T protocol.

Sequence	TA (ms)	FOV (cm)	TR (ms)	TE (ms)	Flip Angle (Degrees)	Voxel Size (mm^3^)	Slices	Acceleration	Other
T1 3D GRE saturated	0:19	24 × 30	6	1.3	9	1.2 × 1.2 × 3	30	Grappa = 3	Saturation method DIXON, breath-holding
T2 SS-FSE	0:55	27 × 30	1500	152	135	0.8 × 0.8 × 2.5	25	Grappa = 2	Parallel saturation bands, free breathing
bSSFP	0:22	30 × 38	500	1.9	46	1.1 × 1.1 × 3	50	Grappa = 2	Offset frequency after scout evaluation, free breathing
IVIM DWI	2:50	38 × 30	4500	67	-	1.7 × 1.7 × 3.5	20	Grappa = 2	B-values: 0, 10, 30, 50, 70, 100, 200, 400, 700, 1000 Avgs: 2, 2, 2, 2, 2, 3, 3, 4, 6, 9 Diffusion mode: 3D diagonal

Example of protocol acquired on Siemens 3T scanners (Vida, Siemens Healthineers, Erlangen, Germany). The examination is performed using the flexible body array combined with the spine array. DWI: diffusion-weighted imaging; FOV: field of view; GRAPPA: GeneRalized Autocalibrating Partially Parallel Acquisition; GRE: gradient echo; IVIM: intravoxel incoherent motion; SS-FSE: single-shot fast spin echo; SSFP: steady-state free precession; TA: acquisition time; TE: echo time; TR: repetition time.

**Table 2:** Suggested 1.5T scanner protocol (readapted from Manganaro et al. [11]).

Sequence	T2-W Haste	T1-W Flash 2D	DWI	True-FISP
TR (ms)	1000	6	5300	3
TE (ms)	119	3	79	1
Slice thickness (mm)	3	3.5	4	4
FOV (mm)	203 × 270	500 × 313	380 × 380	400 × 300
Matrix	256 × 134	256 × 112	192 × 192	256 × 144
Flip angle	150°	10°	90°	60°
Concatenations	1	2	1	1
*b* values (s/mm^2^)	-	-	50,200,700	-
TI (ms)	-	-	185	-
Acquisition time (s)	16–20	30	45	15

DWI: diffusion-weighted imaging; FISP: Fast Imaging with Steady-state Precession; FOV: field of view; GRE: gradient echo; TA: acquisition time; TE: echo time; TI: inversion time; TR: repetition time.

**Table 3:** The potential clinical indications and the role of fCMR.

Potential Clinical Indication	The Role of fCMR
Cardiac malformations	
Anomalous pulmonary venous drainage	-Systemic venous anatomy and connections to the cardiac chambers -Number and normal/abnormal connections of pulmonary veins
Atrial/ventricular septal defect	-Relationship to neighboring structures and outflow tracts -Ventricular size and balance, volumes, function
Tetralogy of Fallot, Transposition of Great Artery, Double outlet right ventricle (TOF, TGA, DORV)	-Ventricular size, and function, better 3D evaluation of cardiac structure relationships -Outflow tracts, great vessel size, and relationships
Hypoplastic left heart syndrome (HLHS)	-Ventricular size and volumes. -Pulmonary parenchymal changes in the presence of a restrictive septum (secondary lymphangiectasia)
Malformation of the great vessels (aortic coarctation, aortic arch anomalies, vascular rings)	-Arch anatomy, branching, dimensions, and flow patterns -Vascular relationships of the great vessels to the bronchopulmonary tree and esophagus (deviation, compression, etc.)
Myocardial anomalies	
Cardiomyopathies	-Myocardial and valvar function -Extracardiac anomalies
Cardiac masses	-Assessment of function, size of mass, and relation to neighboring structures -Potential of tissue characterization -Renal and brain assessment in tuberous sclerosis
Cardiovascular assessment in systemic conditions	
Hydrops fetalis	-Cardiovascular assessment and function
Viral infections	-Cardiovascular status (anatomy, function) -Teratogenic effects of CMV, HIV, toxoplasma, etc.
Heterotaxy and situs	-Thoracic and abdominal visceral situs -Venous anomalies
